# The Role and Applied Value of Mitochondria in Glioma‐Related Research

**DOI:** 10.1111/cns.70121

**Published:** 2024-12-05

**Authors:** Liwen Chen, Hui Zhang, Chao Shang, Yang Hong

**Affiliations:** ^1^ Department of Neurobiology, School of Life Sciences China Medical University Shenyang Liaoning China; ^2^ Department of Neurosurgery, Shengjing Hospital China Medical University Shenyang Liaoning China; ^3^ Department of Urology, Shengjing Hospital China Medical University Shenyang Liaoning China

**Keywords:** glioma, glioma therapy, mitochondria, mitochondrial bioenergetics, mitochondrial dynamics, mitochondrial metastasis, mitophagy

## Abstract

Mitochondria, known as the “energy factory” of cells, are essential organelles with a double membrane structure and genetic material found in most eukaryotic cells. They play a crucial role in tumorigenesis and development, with alterations in mitochondrial structure and function in tumor cells leading to characteristics such as rapid proliferation, invasion, and drug resistance. Glioma, the most common brain tumor with a high recurrence rate and limited treatment options, has been linked to changes in mitochondrial structure and function. This review focuses on the bioenergetics, dynamics, metastasis, and autophagy of mitochondria in relation to glioma proliferation, as well as the potential use of mitochondria‐targeting drugs in glioma treatment.

## Graphical abstract

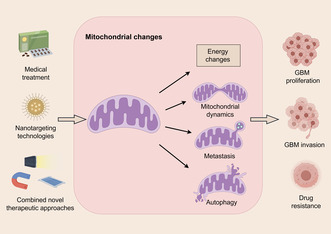

## 1 Introduction

Gliomas are the most common primary intracranial tumors, with glioblastoma (GBM) being the most prevalent, representing approximately 57% of all gliomas and 48% of all primary central nervous system malignancies [1]. At present, the most commonly used methods for treating GBM include surgical resection, radiotherapy, and chemotherapy. In recent years, despite the advancements in various comprehensive treatments, the overall prognosis for patients with GBM remains very poor, with a median survival time of less than 2 years. A study has shown that this may be related to the invasive growth of malignant gliomas in the brain, blood–brain barrier (BBB) limitation, and tumor drug resistance [2]. As a multifunctional organelle [3], mitochondrial disorders can clinically affect multiple systems in the body, especially those tissues or organs that require a significant amount of energy, such as the brain. Current studies have shown that mitochondria can reprogram the metabolism of glioma cells, thereby contributing to the rapid proliferation of glioma cells and enhancing oxidative stress response and resistance to chemical drugs such as Temozolomide (TMZ) [4]. As micron‐scale organelles, most mitochondria function in their intact form after entering the cell, which can be utilized as a drug to a certain extent. At present, mitochondria have been successfully used in treating and researching breast cancer, glioma, melanoma, and other types of cancer. Overall results show that mitochondria can inhibit the growth and proliferation of tumor cells. However, mitochondria are extremely sensitive to ambient oxygen and pH, and they have dual functions in promoting cell survival or inducing cell death in various environments. This environmentally responsive property of mitochondria may contribute to their selective antitumor effects. In addition, the specific molecular mechanisms by which mitochondria play a therapeutic role and other related questions still need to be answered. These include how mitochondria penetrate tissue barriers and the compatibility of cells with exogenous mitochondria. The solution to these questions will be crucial to reveal the mechanism of mitochondrial therapy [5].

Therefore, in this review, we discuss the role of mitochondria in the occurrence and development of glioma, focusing on mitochondrial energy dynamics and recent therapeutic advancements. This review outlines the molecular mechanisms involved in the development of glioma, including mitochondrial metabolic reprogramming, oxidative stress, mitochondrial dynamics, autophagy, and metastasis. It also highlights the significance of mitochondrial research in treating glioma and the potential of targeted therapy for glioma mitochondria. The goal is to provide new strategies for the effective treatment of glioma.

## 2 Changes in Mitochondrial Bioenergetics

Mitochondria in malignant tumor cells are highly active and closely linked to the onset and progression of cancer [6]. Conventional oxygen and nutrient supply cannot meet the physiological needs of cancer cells in solid tumors, which results in significant metabolic stress and metabolic reprogramming within the tumor [7]. Hypoxic conditions in glioma tissues lead to mitochondrial reprogramming in glioma cells to adapt to the unfavorable tumor microenvironment [8]. GBM cells exhibit distinctive energy metabolism characteristics due to their stem cell‐like properties, including low mitochondrial respiration, increased glycolysis for ATP production, and a preference for hypoxia to sustain their stemness and tumor‐forming abilities [9]. The role of mitochondria in metabolic stress and metabolic reprogramming is gradually being revealed.

### 2.1 Reprogramming of Mitochondrial Glucose Metabolism Confers a Rapid Proliferative Advantage in Gliomas

The metabolic center of the cell is the mitochondria, where more than 90% of ATP is produced. Various cancer cells rely on the upregulation of energy production to promote oncogenic potential [10]. Cancer cells utilize large amounts of glucose to produce significant quantities of lactic acid even in the presence of oxygen. This phenomenon is known as the Warburg effect [11], which was one of the first abnormal metabolic pathways identified in cancer. Mitochondrial dysfunction in malignant cells of many cancers, such as GBM, leads to an abnormal reliance on anaerobic metabolism to fulfill energy requirements. The tumor microenvironment determines whether cells primarily rely on glycolysis or oxidative phosphorylation. GBM's reliance on glycolysis may be mainly based on the production of ATP.

**FIGURE 1:**
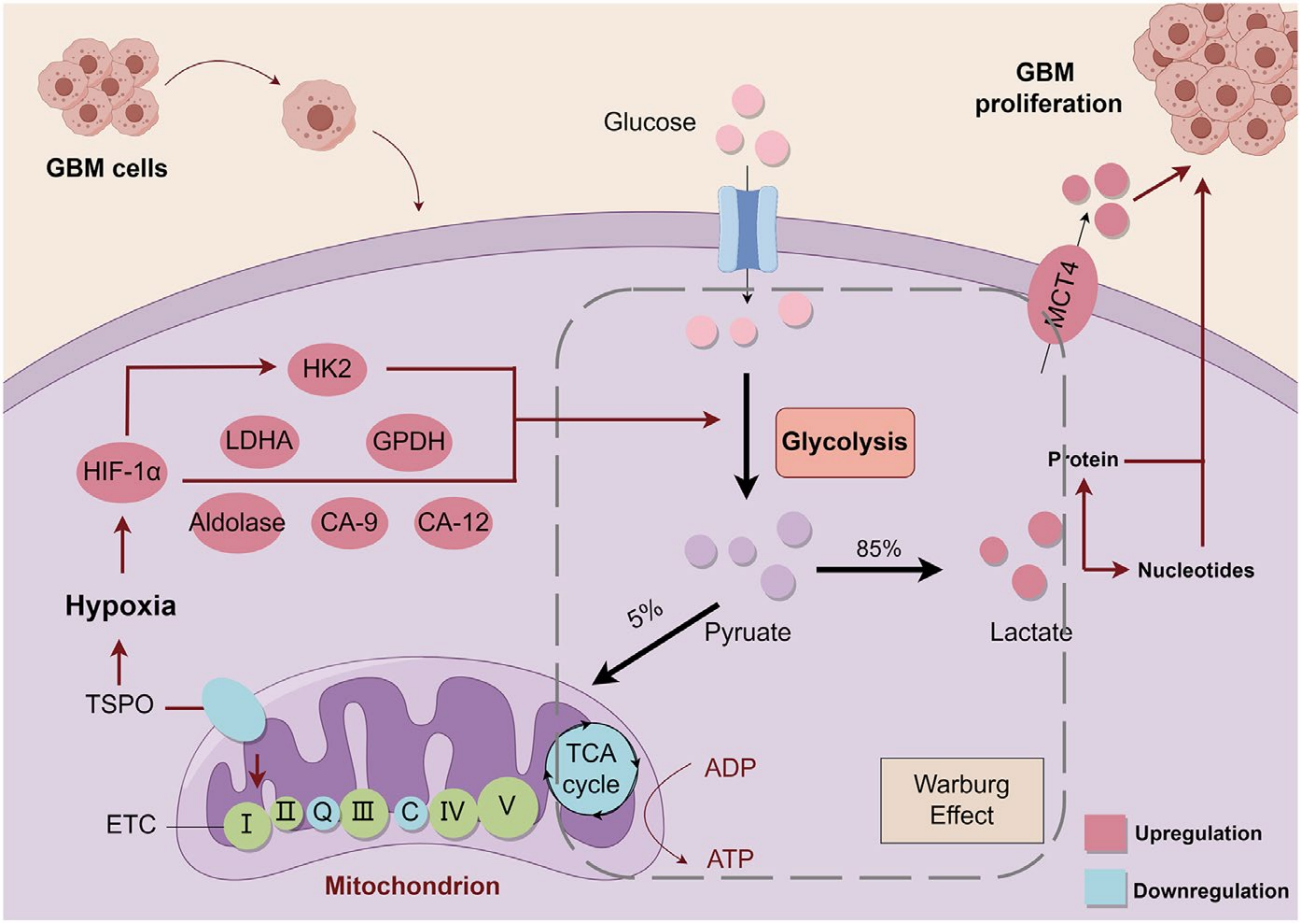
Reprogramming of glucose metabolism for proliferation mechanism in GBM cells. Mitochondrial TSPO deficiency inhibits the mitochondrial respiratory chain, leading to hypoxia. The hypoxia environment activates HIF‐1⍺. HIF‐1⍺ activates HK2, aldolase, LDHA, GPDH, CA‐9, and CA‐12, which promote the conversion of GBM to glycolytic metabolism, and increased lactate levels favor the progression of GBM.

Glycolysis is the most primitive energy pathway, but it is most beneficial in complex cancers such as GBM. Studies have shown that the regulation of glycolysis involves glucose transporter type 1 (GLUT1), hexokinase 2 (HK2), the mitochondrial translocator protein (TSPO), lactate dehydrogenase A (LDHA), hypoxia‐inducible factor 1‐alpha (HIF‐1⍺), the tumor microenvironment, and mitochondrial genes [12]. Compared with low‐grade astrocytomas and normal brain tissue, GBM preferentially expresses HK2, the first enzyme of glycolysis. Increased HK2 expression promotes aerobic glycolysis and inhibits oxidative phosphorylation, which in turn facilitates the rapid proliferation of GBM cells. TSPO is a highly conserved hydrophobic transmembrane protein mainly located in the outer membrane of mitochondria. It plays a crucial role in regulating the metabolic balance between mitochondrial oxidative phosphorylation and glycolysis. Studies have shown that TSPO deficiency induces mitochondrial dysfunction, leading to hypoxia and angiogenesis [13]. This phenomenon is associated with the Warburg effect, a characteristic of cancer cells, where they tend to convert glucose into lactate through aerobic glycolysis, even in the presence of oxygen. When the mitochondrial respiratory chain is inhibited, oxygen cannot function as the terminal electron acceptor in electron transport, resulting in “functional hypoxia.” In this situation, glioblastoma cells upregulate hypoxia‐inducible factor (HIF‐1⍺) to initiate a stress response, activating glycolysis‐related genes and angiogenic factors like VEGF to adapt to the hypoxic environment. This oxygen deficiency triggers a vicious cycle, further exacerbating hypoxia and promoting the glycolytic metabolic shift in GBM. Furthermore, hypoxic conditions in GBM tissues induce several glycolytic enzymes and glucose transporter proteins to coordinate tumor cell adaptation to metabolic stress. The transcription factor HIF‐1⍺ is activated in hypoxia and promotes the expression of several metabolic proteins, such as HK2, leading to a metabolic shift toward glycolysis [14]. HIF‐1⍺ also activates aldolase, glyceraldehyde‐3‐phosphate dehydrogenase (GPDH), LDHA, plasma membrane lactate transporter protein, carbonic anhydrase 9 (CA‐9), and carbonic anhydrase 12 (CA‐12). These molecules can stimulate the glycolytic pathway and facilitate the transfer of lactate into the extracellular space. Lactate in GBM is more than a metabolic byproduct; it promotes tumor proliferation, invasion, and poor prognosis. Through autocrine and paracrine signaling, lactate activates receptors like GPR81 and the HIF‐1⍺ pathway, boosting glycolysis and energy production [15]. It suppresses immune responses by inhibiting T and NK cells, while promoting M2 macrophages that support tumor growth [16, 17]. Lactate also acidifies the tumor microenvironment, increasing MMP activity to aid invasion and metastasis [18]. Additionally, it enhances resistance to radiotherapy and chemotherapy, and protects tumor cells from oxidative stress by upregulating antioxidants [19]. It has been found that lactate may act in a cytokine‐like manner by binding to membrane receptors, and high lactate levels may be associated with a poor prognosis of GBM [20]. In conclusion, the substantial amount of lactate produced by glycolysis affects the microenvironment in several ways that promote the progression of GBM. Moreover, the mechanism of glucose metabolism reprogramming for GBM cell proliferation is illustrated in Figure 1.

In summary, metabolic reprogramming in GBM cells downregulates the TCA cycle and OXPHOS pathways, leading to a reliance on glycolysis as the primary energy source, rather than as an oxidative metabolic pathway. Although glycolysis produces less ATP than phosphorylation, the carbon in glucose can be used to produce other essential substances for cellular replication. Additionally, the large amount of lactic acid produced by glycolysis is also beneficial for the progression of GBM development. Therefore, mitochondrial metabolic reprogramming confers the advantage of rapid proliferation of GBM.

### 2.2 Mitochondrial Glutamine Metabolism Provides Energy for Glioma Cell Growth

Glutamine is an amide of glutamic acid, a nonessential amino acid for mammals that is considered an important metabolite for tumor cell growth. It is also a major substrate for DNA and fatty acid synthesis [21]. To meet the growth requirements of tumors in unfavorable microenvironments, ATP production from glycolysis is not sufficient. Therefore, tumor cells exhibit an increased dependence on other nutrients to satisfy the mitochondrial TCA cycle, usually relying on glutamine. This is because glutamine is the most abundant free amino acid in the circulation and cells, a phenomenon known as “glutamine addiction” [22]. There is now growing evidence that glutamine metabolism is a hallmark of GBM [11, 23]. Glutamine primarily energizes GBM cells with mesenchymal molecular subtype [24].

Furthermore, there is evidence indicating that glutaminolysis facilitates the generation of high‐energy phosphates in tumor cells that have dysfunctional glycolytic pathways. Glutamine‐derived succinate provides enough ATP through mitochondrial substrate‐level phosphorylation to sustain GBM growth when oxidative phosphorylation is turned off [25, 26]. For example, the T98G cell line supports high‐energy phosphate production through the sequential conversion of glutamine → glutamate → α‐Ketoglutaric acid (α‐KG) → succinyl‐CoA → succinate [25]. Increased glutamine metabolism is another common alteration in tumor cells. During hypoxia or mitochondrial dysfunction, glutamine‐produced α‐KG is converted to citrate in a reductive carboxylation reaction catalyzed by IDH2. The newly generated citric acid will be excreted from the mitochondria into the cytoplasm. This process produces acetyl‐CoA, which promotes fatty acid and amino acid production. Additionally, it generates the reductant reduced nicotinamide adenine dinucleotide phosphate (NADPH) [27], providing advantages for the rapid proliferation of glioma cells.

Several studies have shown that the release of excitotoxic concentrations of glutamate through cystine–glutamate antitransporter proteins promotes the growth of malignant GBM. When N‐methyl‐D‐aspartate receptors are activated for an extended period in nearby neurons, they trigger an influx of Ca^2+^ into the cells, resulting in apoptosis. The death of cells around a tumor is believed to make it easier for the tumor to spread into the surrounding tissues and allow malignant cells to better absorb nutrients, giving them a competitive edge [28].

In brief, glutamine metabolism provides energy for GBM growth. Glutamine metabolism provides a pathway for glioma cell proliferation to generate large amounts of energy. When the glycolysis pathway is impaired, succinic acid derived from glutamine can supply a significant amount of energy for glioma cell activities by undergoing phosphorylation at the mitochondrial substrate level, thereby sustaining GBM growth.

### 2.3 Mitochondrial Fatty Acid Metabolism is a Key Adaptive Response of Gliomas to Deleterious Conditions

Fatty acids (FA) are compounds that contain three elements: carbon, hydrogen, and oxygen. In the brain, fatty acids account for 50%–60% of the dry weight of the entire brain, and they are involved in many physiological activities, such as neurotransmitter transport, myelination, and axonal growth [29]. In mammalian cells, fatty acid oxidation (FAO) mainly consists of α‐oxidation and β‐oxidation. FA can undergo β‐oxidation after α‐oxidation [30]. The process of fatty acid β‐oxidation involves the activation of FA, the transfer of acyl‐CoA, and the β‐oxidation of acyl‐CoA. Normal fatty acid metabolism contributes to the orderly conduct of life activities, but dysregulated fatty acid metabolism is associated with severe central nervous system pathology [31]. Studies have shown that GBM is dependent on FAO, with nearly 80% of oxygen respiration relying on it [29]. In addition, GBM cells exhibit specific alterations in different aspects of lipid metabolic reprogramming, which, in turn, affects the availability of structural lipids for membrane synthesis, representing a crucial adaptation in response to harmful conditions [32].

Changes in fatty acid β‐oxidation are key factors in malignant GBM [33]. Studies have shown that FAO has both FA synthesis (FAS) and catabolic FA roles, enabling GBM cells in hypoxic or nutrient‐depleted regions to synthesize and consume FA to produce ATP for survival [29]. Increased FAS is a hallmark of cancer [27]. Factors related to FA synthesis are upregulated in GBM, such as the epidermal growth factor receptor (EGFR) signaling pathway, which is central to FA synthesis in GBM. Thus, GBM with EGFR variant III (EGFRvIII) amplification can exhibit higher proliferation rates due to increased de novo lipogenesis and overall metabolic activity [34]; increased expression of fatty acid synthase (FASN) in GBM cells promotes FA synthesis, thereby enhancing GBM cell migration and recurrence [35]; the upregulated expression of ATP‐citrate lyase (ACL) in GBM significantly promotes fatty acid and cholesterol synthesis to supply nutrients for GBM cell proliferation [36].

**FIGURE 2:**
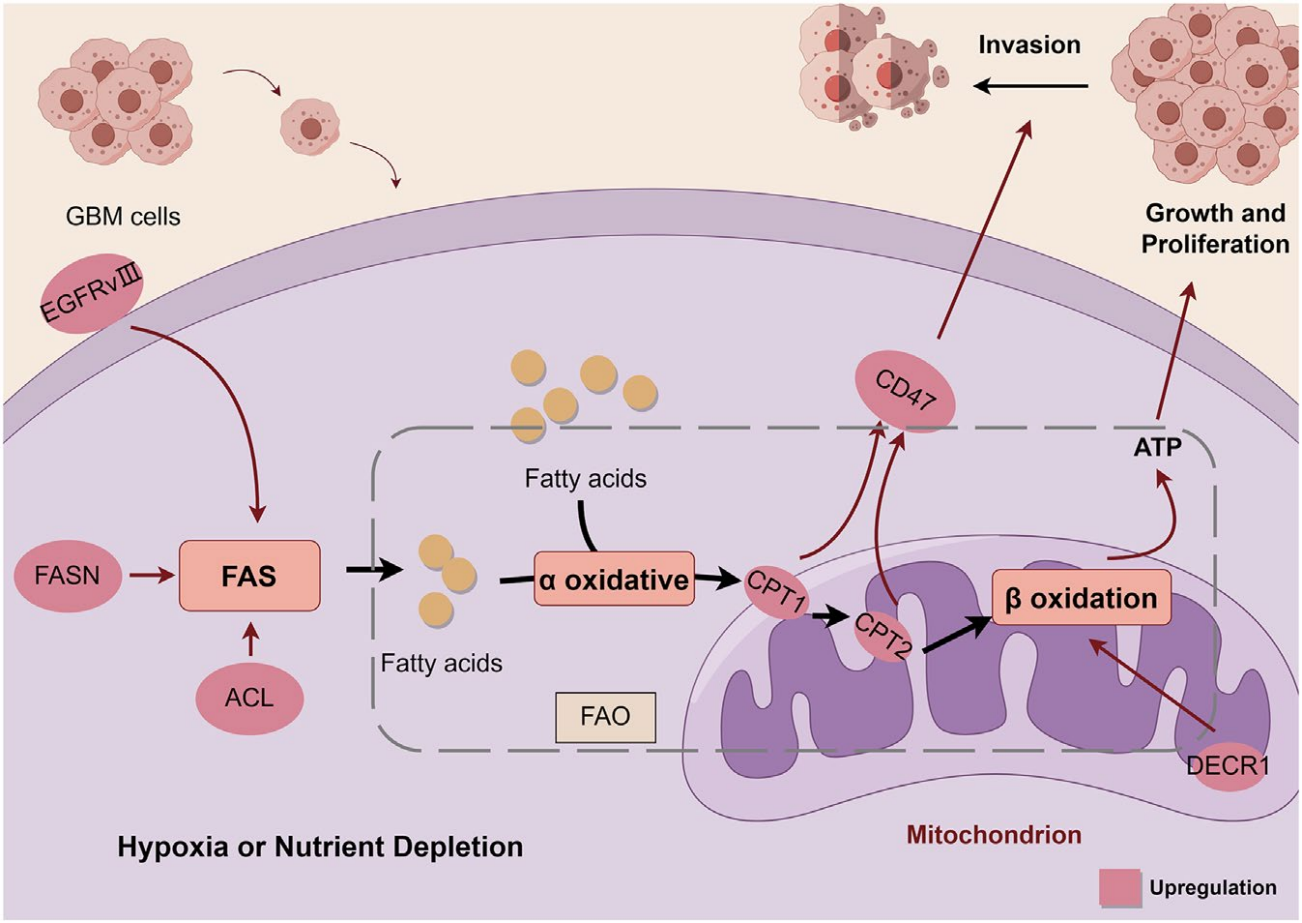
Mechanisms of fatty acid metabolism promoting proliferation and invasion in GBM cells. Fatty acid metabolism consists of FAS and FAO. Overexpression of EGFRvIII, FASN, and ACL promotes FAS. FAO consists of α‐oxidation and β‐oxidation, and FA undergoes α‐oxidation and enters the mitochondria for the β‐oxidation process. CPT1, CPT2, and DECR1 overexpression promotes β‐oxidation. Enhanced expression of CPT1 and CPT2 promotes invasive growth of GBM through CD47. β‐oxidation releases a large amount of energy to support GBM growth and proliferation.

Many of the enzymes necessary for the fatty acid β‐oxidation process are plentiful in GBM cells, allowing them to easily acquire ATP by oxidizing fatty acids to promote cell proliferation. Studies have shown that mitochondrial FAO‐related enzymes including carnitine palmitoyltransferase 1 (CPT1), carnitine palmitoyltransferase 2 (CPT2), sterol carrier protein 2 (SCP2), 2,4‐dienoyl‐CoA reductase (DECR1), and acyl‐CoA‐binding proteins, were significantly upregulated in GBM [37]. Among them, SCP2 is a lipid transfer molecule that ensures an adequate supply of lipids in mitochondria [38]. DECR1 plays a crucial role in mitochondrial lipid β‐oxidation and the metabolism of polyunsaturated fatty acid allyl‐CoA esters. CPT1 and CPT2 proteins are elevated in patients with recurrent GBM and poor prognosis. Enhanced TAO promotes invasive growth of GBM through integrin‐related protein (cluster of differentiation 47, CD47) mediated immune evasion [39]. The neural stem cell pro‐proliferative factor acetyl‐CoA‐binding protein is highly expressed in GBM, and by binding to acetyl‐CoA, which participates in FAO in multiple preclinical models, it maintains a high proliferation rate of GBM cells, promotes tumor growth, and reduces survival in GBM patients [40]. Figure 2 illustrates the mechanisms of fatty acid metabolism that promote proliferation and invasion in GBM cells.

Furthermore, an imbalance in FAO has emerged as a contributing factor to TMZ resistance in GBM cells. Studies have shown that mutations in FAS and oxidative metabolism are closely associated with rapid proliferation, migration, and resistance to radiotherapy in the GBM [41, 42]. However, there are still many unanswered questions in the field of FA anabolism in GBM, and considerable efforts should be made to unravel these complex processes.

### 2.4 Others

The urea cycle is a crucial biochemical process responsible for converting the toxic metabolic byproduct ammonia into urea. This process plays a significant role in the metabolism of nitrogenous waste and in biochemical changes associated with cancer. Arginine is considered a substrate that upregulates many metabolic functions in cancer cells. Elevated arginine utilization is observed in GBM under physiological conditions. Maintaining an adequate intracellular source of arginine depends on uptake from the extracellular environment [28]. Interestingly, certain phenotypes of GBM show a reduced capacity for endogenous arginine synthesis, which can be effectively inhibited by silencing the rate‐limiting enzyme argininosuccinate synthase 1. This inhibition manifests as nutritional deficiencies in GBM patients. This further promotes its dependence on extracellular arginine.

In glioblastoma multiforme, one‐carbon metabolism (OCM) is a crucial pathway for sustaining biosynthetic activity. OCM encompasses a series of biochemical reactions occurring in the mitochondria and cytoplasm, involving the folate and methionine cycles. These reactions provide essential methyl groups, facilitating the synthesis of key metabolites such as phospholipids, amino acids, and DNA. As a crucial element of GBM metabolism, the targeted inhibition of methylenetetrahydrofolate dehydrogenase 2, a key enzyme in the one‐carbon metabolism of folate, effectively induces apoptosis and leads to a significant reduction in the invasive potential of GBM cells. Conversely, the addition of exogenous methionine to GBM cells stimulates and sustains the growth of this cell [43].

## 3 Mitochondrial Oxidative Stress Promotes Glioma Development in Multiple Ways

Oxidative stress is a condition characterized by an imbalance between oxidative and antioxidant processes in the body. This imbalance results in the infiltration of neutrophils, heightened secretion of proteases, and the generation of substantial quantities of oxidative intermediates. Reactive oxygen species (ROS) are the main cause of oxidative stress [44]. Due to the high metabolism of tumor cells, the redox potential of mitochondria is altered, causing NADPH oxidase to the overproduction of ROS [45]. One of the characteristics of tumor cells is high levels of ROS. The brain is particularly susceptible to the destructive effects of ROS due to its high metabolic activity and relatively limited cellular regenerative capacity [46].

Studies have shown that high intracellular ROS disrupts the mitochondrial membrane potential (MMP) of GBM cells, leading to defects in the mitochondrial electron transport chain and reduced metabolic oxygen consumption. This results in oxidative stress, enabling GBM cells to accelerate proliferation and enhance invasiveness [47]. Aquaporin 8 (AQP8), which is overexpressed in GBM cells, protects glioma cells from high redox levels to a certain extent by increasing ROS levels, which facilitates the proliferation and growth of GBM cells [48]. In addition, high levels of intracellular ROS may also be expelled from the cells, such as H_2_O_2_ through aquaporin proteins. Hydrogen peroxide may alter the cell cycle phases, leading to GBM progression [49].

High levels of ROS can also promote the occurrence, progression, and resistance to treatment of GBM by damaging DNA, leading to the accumulation of mutations and genomic instability, as well as the reprogramming of cell metabolism and signaling [50, 51]. For example, mutations in the nicotinamide adenine dinucleotide phosphate‐dependent homodimer enzyme IDH1/2 in GBM lead to NADPH‐dependent conversion of α‐ketoglutarate into D‐2‐hydroxyglutarate (D‐2HG). D‐2HG induces oxidative stress in rat brain cells and enhances tumorigenesis [52]. Additionally, overexpression of EGFRvIII in GBM results in elevated levels of ROS and alterations in the genome of GBM cells. The rate of change is high [49]. In addition, ROS accumulation not only directly damages DNA by increasing cellular mutations or enhancing oncogenic phenotypes but also indirectly acts as a secondary messenger in intracellular signaling cascades [49, 53]. Recent studies have found that oxidized cancer cells in GBM subtypes exhibit a phenomenon called the “Reverse Warburg Effect” [54, 55]. This effect involves GBM cells secreting H_2_O_2_ into the tumor microenvironment to induce oxidative stress in adjacent stromal cells. This process leads to an increase in nutrients and ATP, providing energy that enhances the proliferation ability of GBM cells.

ROS are regulators of cell migration and invasion [56]. ROS levels are increased in glioma cells, leading to the upregulation or downregulation of numerous functional genes that are crucial in glioma angiogenesis and tumor cell migration or invasion. These genes are also implicated in the abnormal activation of redox‐sensitive signaling pathways related to tumor invasion and migration [57]. Liang et al. found that overexpression of the eIF4E gene in GBM cells promoted ROS production and enhanced the proliferation, invasion, and migration of U251 cells [58]. ROS promotes the activation of focal adhesion kinase (FAK) and proline‐rich tyrosine kinase 2 (Pyk2), leading to the migration and invasion of glioma cells [57, 59]. In addition, matrix metalloproteinase‐9 (MMP‐9) induced by ROS‐activated extracellular signal‐regulated kinases is involved in the invasion and migration of the U87MG cell line [60]. Recent experiments have identified cells with high motility potential and metabolic specificity. These cells are characterized by enhanced mitochondrial loading, oxidative stress, and mobilization of the cysteine‐metabolizing enzyme 3‐mercaptopyruvate sulfotransferase (MPST). GBM cells depend on this enzyme due to enhanced ROS production and MPST activity to enhance its movement [61]. The proximity of ROS molecules also has the potential to disrupt cell membranes, supporting tumor growth and invasion [50]. Li et al. found that the overexpression of NADPH oxidase subunit 4 (Nox4) in GBM cells stimulated an increase in ROS and promoted GBM invasion and associated angiogenesis [62]. ROS also promotes glioma cell metastasis by increasing vascular permeability [56]. ROS phosphorylates heat shock protein 27 by activating p38, causing dynamic changes in endothelial cell actin, and promoting the tumor cell invasion process [63, 64].

**FIGURE 3:**
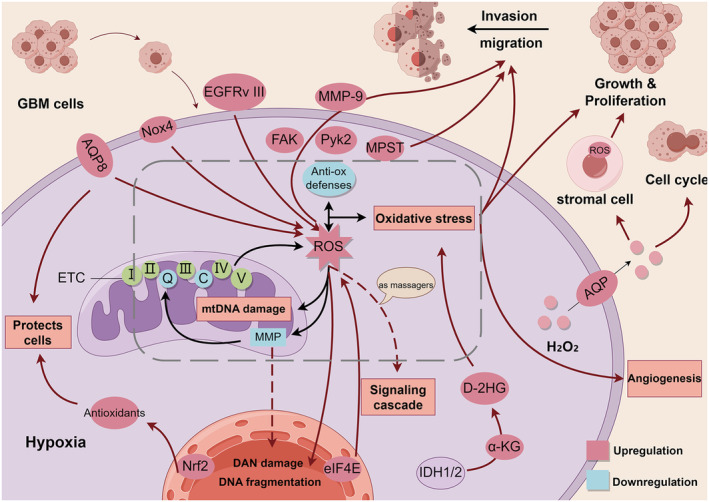
Oxidative stress in GBM cells. Changes in intracellular mitochondrial redox potential lead to high levels of ROS production, and oxidative stress occurs as a result of an imbalance between intracellular ROS and antioxidant factors. High levels of ROS disrupt mitochondrial DNA(mtDNA), nuclear genome, and MMP. Overexpression of AQP8, EGFRvIII, Nox4, and eIF4E genes promotes ROS production. High levels of ROS promote the activation of FAK, Pyk2, and MMP‐9 to promote cell migration and invasion. ROS and MPST can work together to promote cell migration. ROS act as secondary messengers in the intracellular signaling cascade in the GBM cells. IDH1/2 mutations result in the conversion of α‐KG to D‐2HG triggering oxidative stress. Oxidative stress promotes cell growth and proliferation, migration and invasion, and angiogenesis. Nrf2 regulates the production of antioxidants to protect cells from damage caused by oxidative stress. Water channel proteins drain H_2_O_2_ into the tumor microenvironment to regulate the cell cycle and induce the production of nutrients and ATP by proximal stromal cells to support GBM cell proliferation.

The ROS is a “double‐edged sword” for tumors. A certain level of ROS promotes tumor development, while excessive ROS has cytotoxic effects, inducing activation of apoptosis and autophagy pathways [44, 50, 65]. Therefore, the removal of excess ROS is also essential for glioma cell metabolism. The nuclear factor‐erythroid 2‐related factor 2 (Nrf2) is a key regulator of the antioxidant response. In response to ROS overproduction, Nrf2 translocates into the nucleus and regulates the expression of endogenous genes. The transcription products of these genes act as antioxidants to avoid cell death caused by oxidative stress [66]. In short, high levels of ROS are one of the characteristics of GBM. Unusual levels of ROS lead to increased oxidase activity and decreased reductase activity, disrupting the normal redox metabolic pathways of the cell. At the same time, the presence of ROS causes DNA mutations and signaling alterations in multiple ways that promote glioma development, increasing the proliferation and invasiveness of gliomas. An overview of oxidative stress in GBM cells is depicted in Figure 3.

## 4 Mitochondrial Dynamics Have Multiple Roles in GBM Proliferation and Invasion

Mitochondrial dynamics undergo continuous processes of fission, fusion, autophagy, and metastasis, which regulate the distribution of mitochondria within the cell and the remodeling of mitochondrial structure and function. Mitochondria are the “power plants” of the cell. The fusion and fission of mitochondria maintain their normal shape, distribution, and function, and play a crucial role in maintaining normal physiology, metabolism, and cell development. Mitochondrial fusion allows gene products to be transferred between mitochondria for optimal function, especially under metabolic and environmental stresses [67]. Mitochondrial fission is essential for mitochondrial division and quality control [68]. The main regulators of mitochondrial fusion are mitofusin (Mfn) proteins and optic atrophy‐1 (OPA1) proteins, with Mfn containing two isoforms, Mfn1 and Mfn2 [69]. Mitochondrial fission is mainly regulated by dynamin‐related protein 1 (DRP1) [70]. Imbalances in mitochondrial fusion and fission are usually closely associated with the development of tumors in organisms, including GBM.

Reduced mitochondrial fusion has pro‐tumor effects. Many studies have shown that reduced levels of Mfn in human lung, liver, and breast cancers are associated with worse survival outcomes. Tumor cell lines with silenced Mfn2 display higher viability and invasiveness [71, 72, 73]. However, the role of Mfn in GBM has not been reported, and lower levels of OPA1 mRNA are significantly associated with GBM shorter survival time [74]. It was found that the deletion of OPA1 in GBM could promote GBM cell invasion [74], indicating that OPA1 is a vital factor in GBM invasion. In the study by Luo et al. [75], they found that overexpression of carnitine palmitoyltransferase 1A can promote mitochondrial fusion and prolong the survival time of GBM tumor‐bearing mice. The activity of nuclear respiratory factor 1 is associated with the invasiveness of GBM, leading to poor prognosis and resistance to TMZ treatment [76]. However, OPA1 molecules are cleaved by various mitochondrial proteases, resulting in the formation of different cleaved isoforms: the OPA1 short protein (S isoform) and the long protein (L isoform). Among them, the S isoform mainly induces mitochondrial fission, and the L isoform mainly mediates mitochondrial fusion [77, 78]. This indicates that OPA1 can activate various isoforms that cannot be produced through cleavage by proteases in response to different environmental stimuli, leading to entirely distinct forms. In an experiment to investigate the subcellular mechanism of sulforaphane cysteine (SFN‐Cys) inhibiting human GBM invasion, Zhou et al. found that SFN‐Cys significantly downregulated S isoform OPA1, thereby inhibiting GBM invasion [79].

Increased mitochondrial fission is a pro‐tumorigenic phenotype [80]. DRP1 has been observed to have enhanced expression in GBM and is involved in maintaining GBM stem cell properties, such as initiating and maintaining tumor characteristics, as well as promoting migration and invasiveness, which are core factors in GBM [81]. Studies have shown that for the highly aggressive GBM, as it progresses, the central hypoxic zone expands, leading to cell necrosis. Hypoxia in the surrounding area of necrosis can be induced by regulating mitochondrial dynamics. Increased DRP1 mRNA and protein levels promote mitochondrial fission, which is essential for migration and invasion events, thereby enhancing the stemness of GBM [82, 83]. HIF‐1, which plays a key role in the hypoxic process, can upregulate DRP1 expression and facilitate cell migration to aerobic areas. Thus, DRP1 can induce human U251MG cell migration under hypoxic conditions [83]. At the same time, the disrupted‐in‐schizophrenia 1 (DISC1) protein in the mitochondria of GBM cells can also enhance the expression of DRP1 and participate in cell migration [84]. The expression of tumor necrosis factor receptor‐associated protein 1 (TRAP1) is also increased in glioma cells, which enhances DRP1‐mediated mitochondrial fission and GBM cell migration [85], Ras‐related protein Rab32 can regulate ERK/DRP1 pathways [86], promotes mitochondrial fission, thereby promoting the mesenchymal transformation of GBM and promoting the migration and invasion of tumor cells.

The progression of the cell cycle and the coordination of mitochondrial dynamics are tightly linked. For instance, mitochondrial fusion occurs when the cell enters the G1 phase, while mitochondrial fission occurs when the cell enters the S phase. In the study by Wang et al. [87], Krüppel‐like factor 4 (KLF4) induces U87MG cells to enter the S phase earlier and blocks them in the G2/M phase. The study also found that KLF4‐induced mitochondrial fusion directly leads to KLF4‐induced cytoprotection and alterations in the cell cycle. Therefore, mitochondrial fusion plays a crucial role in regulating the cell cycle of U87MG cells. Mitochondrial dynamics are closely related to cellular metabolism. For example, Mfn2, OPA1/3, and DRP1 are involved in the auxiliary control of mitochondrial energy production [88]. Overexpression of Mfn2 increases the ability of mitochondria to produce ATP and stimulate glucose oxidation [89]. In the absence of OPA1, the ultrastructure of mitochondrial cristae is severely damaged, leading to a reduction in oxygen consumption rate and ATP levels. Respiratory supercomplex organization is lost, and complex V assembly is incomplete [90, 91]. Wang et al. found that KLF4 also increased spare respiratory capacity and reactive oxygen species in GBM cells, implying that mitochondrial fusion enhances the respiratory capacity of GBM cells [87].

**FIGURE 4:**
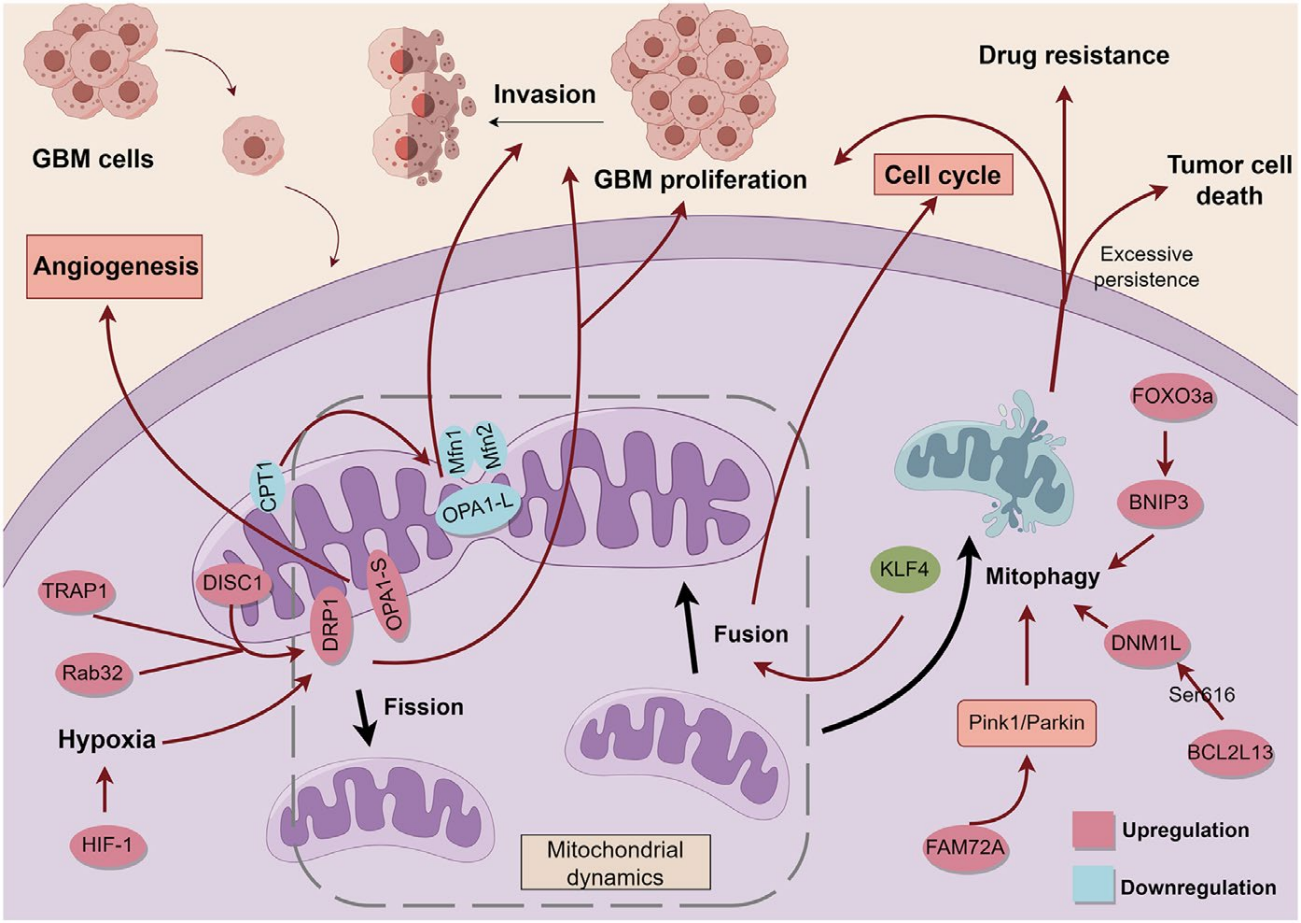
Mitochondrial dynamics and mitophagy in GBM cells. Mitochondrial fusion‐associated proteins Mfn1, Mfn2, and OPA1‐L isoform (OPA1‐L) were expressed at reduced levels, and a reduction in CPT1 protein induced low expression of OPA1‐L, which reduced mitochondrial fusion and thus promoted cell invasion. KLF4 protein affects mitochondrial fusion and thus the cell cycle. The overexpression of DISC1, TRAP1, Rab32 protein, and HIF‐1 induces a hypoxic environment that promotes DRP1 protein‐mediated mitochondrial fission, which facilitates cell proliferation and invasion. overexpression of DPR1 and OPA1‐S isoform promotes angiogenesis. FAM72A regulates mitochondrial fusion through the Pink1/Parkin signaling pathway. Pink1/Parkin signaling pathway to regulate mitophagy, thereby promoting glioma progression. Targeting of DNM1L at the BCL2L13 Ser616 locus resulted in altered high mitochondrial autophagic fluxes, which significantly promoted GBM cell proliferation and invasion. FOXO3a generates drug resistance by promoting BNIP3‐mediated mitophagy.

In addition to the above, mitochondrial dynamics play an important role in tumor angiogenesis. The proteins OPA1 and DRP1, which are associated with mitochondrial dynamics, have recently been reported as important players in tumor angiogenesis [92, 93]. OPA1 promotes angiogenesis by influencing NFκB activity and the expression of angiogenic genes. It is crucial for tumor growth and metastasis [92]. Li et al. found that celastrol inhibits angiogenesis by downregulating OPA1, indicating that OPA1 expression is associated with angiogenesis [94]. Kim et al. found that DRP1 induced endothelial histiocyte‐mediated angiogenesis under hypoxic conditions [93]. However, the roles of mitochondrial dynamics in glioma angiogenesis are unclear, and further studies are needed to confirm their significance. The mitochondrial dynamics in GBM are summarized in Figure 4.

In summary, decreased mitochondrial fusion and increased mitochondrial fission have a pro‐glioma proliferative, migratory, and invasive effect. Different isoforms of OPA1 have contrasting effects on glioma development. Mitochondrial dynamics play crucial roles in cell metabolism, cell cycle regulation, and angiogenesis, impacting glioma development significantly. However, there are few reports in the relevant literature, and further studies are needed to fully elucidate the relationship between mitochondrial dynamics and GBM progression. As research in this area progresses, focusing on mitochondrial dynamics will become an effective strategy for glioma therapy.

## 5 Mitochondrial Autophagy Plays a Dual Role in Glioma Development

Mitochondrial autophagy (mitophagy) plays a crucial role in regulating mitochondrial function and signaling pathways, and it may contribute to the progression of GBM. Family with sequence similarity 72 member A (FAM72A) is significantly upregulated in gliomas, significantly correlated with WHO grades, and associated with poor clinical outcomes. FAM72A has been shown to regulate mitophagy through the Pink1/Parkin signaling pathway, thereby promoting the progression of glioma [95]. In addition, mitophagy can also enhance the plasticity of glioma stem cells (GSC) by regulating mitochondrial function, enabling them to better adapt to the tumor microenvironment. Methyltransferase‐like 3 (METTL3) overexpression inhibits mitophagy in GSCs. Low expression of METTL3 in GSCs increases mitochondrial membrane potential and maximum respiratory capacity by regulating mitochondrial function through mitophagy. This process promotes GSC proliferation and renewal [96], contributing to glioma development.

Alterations in mitophagy flux may play a crucial role in cancer progression and metastasis [97]. BCL2‐like 13 (BCL2L13) is a member of the BCL2 family that regulates cell growth and apoptosis in various types of tumors. Zhang et al. discovered that targeting the dynamin 1‐like (DNM1L) protein at the BCL2L13 Ser616 locus in GBM resulted in a significant increase in mitophagy flux, ultimately promoting the proliferation and invasion of GBM cells [98]. Mitophagy also occurs to enhance drug resistance in glioma cells. He et al. found that Forkhead box transcription factor 3a (FOXO3a) attenuated TMZ‐induced DNA double‐strand breaks in human glioma cells by promoting Bcl‐2/adenovirus E1B 19‐kDa‐interacting protein 3 (BNIP3) mediated mitophagy [99].

However, mitophagy is also a double‐edged sword in tumor cells [96]. In addition to the aforementioned effects on promoting tumor proliferation, invasion, and drug resistance, excessive or sustained autophagy may induce tumor cell death, particularly in apoptosis‐deficient cancer cells [100]. In recent years, drugs targeting the mitophagy pathway can promote GBM cell death by inducing mitochondrial damage, impairing ATP synthesis, and depleting significant amounts of ATP. Induction of lethal autophagy has emerged as a strategy for eliminating GBM cells and has been reported to be an effective method for eradicating cancer cells [101, 102, 103]. The mitophagy in GBM is shown in Figure 4.

## 6 Mitochondrial Transfer is Involved in the Progression of Glioma

Mitochondrial transfer is a process that involves the transfer of mitochondria or their genes into recipient cells. In cancer pathology, cancer cells can acquire mitochondria from neighboring noncancerous cells, which not only replenishes the number of mitochondria but also leads to the acquisition of tumor‐specific phenotypic characteristics, such as increased proliferation rate, migration, invasion ability, and increased resistance to chemotherapeutic drugs [104]. Mitochondrial transfer mechanisms can be divided into three categories [105]: (1) Formation of transient cell connections through which mitochondria can move from one cell to another; (2) Ejection of mitochondria into extracellular vesicles for delivery to recipient cells; and (3) Release of free mitochondria for capture by recipient cells.

First, nanotubes and tumor microtubules are transient long‐distance membrane channels formed between cells that allow mitochondria to move between different cells. In GBM, the formation of TNTs and TMs promotes the delivery of tumor‐derived mitochondria, increases oxidative phosphorylation and ATP production, and enhances the proliferation rate and invasiveness of GBM cells [106]. TNTs and TMs also enhance resistance to chemotherapy by establishing networks between tumor cells, especially the abundant connections of TMs that increase tumor resistance to the chemotherapeutic drug temozolomide [107, 108]. Secondly, extracellular vesicles are small vesicles secreted by cells that can carry mitochondria or their fragments and transfer them to other cells, thereby affecting the function of the recipient cells. This mechanism is particularly prominent in the tumor microenvironment. Glioma cells transfer mitochondria to tumor‐associated macrophages through vesicles, changing the function of immune cells and inhibiting their antitumor ability [109]. The third major mechanism of mitochondrial transfer is the release of free or naked mitochondria, which are then captured by recipient cells. The capture of free mitochondria is considered to be one of the means by which tumor cells escape adverse conditions (such as hypoxia or chemotherapy). Glioma cells improve their damaged mitochondrial function by capturing exogenous free mitochondria, thereby improving their antioxidant capacity and energy supply. For example, one study pointed out that glioma cells can obtain free mitochondria from neighboring astrocytes or other cells in the microenvironment. This transfer not only promotes the survival of tumor cells but also may increase their invasiveness and drug resistance [105, 110]. In addition, other studies have shown that the capture of free mitochondria may also affect the growth of gliomas by regulating the immune response in the tumor microenvironment. For example, tumor‐associated macrophages may participate in regulating tumor immune escape mechanisms through the transfer of free mitochondria, making tumors more invasive and treatment‐resistant [111].

## 7 The Impact of the Interaction Between Mitochondria and Nuclear Genes on Glioma

Mitochondrial genetics plays an important role in glioma. Mutations in mtDNA often affect the respiratory chain complexes of cells, especially complexes I and IV, leading to impaired oxidative phosphorylation and excessive production of ROS. These ROS not only disrupt the normal function of cells but also activate pro‐oncogenes in the nucleus, further promoting the malignant growth of tumors [112]. In addition, the mitochondrial DNA repair mechanism in glioma cells, especially the base excision repair pathway, often has weakened function. This repair disorder leads to an increase in the mtDNA mutation rate, making it easier for cells to adapt to stressful environments and accumulate more gene mutations, thereby promoting the progression of glioma [110, 112].

The interaction between mitochondria and nuclear genes is also crucial for the occurrence and development of glioma. Mitochondrial dysfunction can interfere with the normal regulation of nuclear genes through ROS signals, activate multiple pro‐oncogene pathways, such as the NF‐κB signaling pathway, and lead to enhanced proliferation and invasion of tumor cells. Studies have shown that this nuclear‐mitochondrial signaling imbalance plays a promoting role in the malignant development of glioma [111]. Therefore, regulating the function of mitochondria and its interaction with nuclear genes may become a new direction for the treatment of glioma in the future.

## 8 Application of Mitochondria in the Treatment of Gliomas

Drug therapy is the most common treatment for GBM besides surgical resection. Mitochondria are very important organelles in tumor cells, playing a central role in ATP production. They are also involved in a variety of cellular processes, such as cell metabolism, proliferation, and cell death [113]. GBM cells that rely on glycolytic metabolism easily adapt to bioenergetic stress by activating mitochondrial pathways to ensure survival and grow [114]. All changes in mitochondrial dynamics in GBM cells contribute to the proliferation, invasion, and progression of glioma cells. Furthermore, varying levels of GBM mitophagy can have distinct impacts on GBM progression. Therefore, targeting mitochondria in GBM is a promising therapeutic strategy [115].

### 8.1 Drug Combination Strategies and Treatment Mechanisms

A growing number of studies have demonstrated that combining two or more drugs leads to a more significant antitumor effect. Additionally, multiple drugs can be used in combination with TMZ to treat gliomas. Metformin was found to inhibit the expression of O6‐methylguanine methyltransferase in TMZ‐resistant glioblastoma cells. This inhibition can induce a synergistic antitumor response in glioma cell lines by increasing cell death [116, 117]. In addition, Resveratrol, Quercetin, Berberine, and Curcumin can all be used in combination with TMZ to effectively inhibit cell proliferation and induce apoptosis, achieving anti‐GBM effects [118]. The combination of Cannabidiol and TMZ showed significant synergistic effects in controlling tumor size and improving survival in patient‐derived neurosphere cultures and mouse in situ models [100]. Delong et al. found that the combination therapy of TMZ (250 μM) + celecoxib (30 μM) exhibited significant inhibitory effects on glioblastoma LN229 and LN18 cell lines [119].

Furthermore, the concurrent use of other medications will also amplify the antitumor effect. For example, Trang et al. found that histone deacetylase inhibitors synergized with ONC201 to treat GBM, promote intrinsic apoptosis, and improve survival in an in situ GBM xenograft model [120]. Han et al. found that the combination of PENAO and sodium dichloroacetate inhibited cell proliferation and induced mitochondria‐mediated apoptosis in GBM. Furthermore, sodium dichloroacetate enhanced PENAO‐induced oxidative damage by inhibiting glycolysis, thereby reducing PENAO‐induced acid production. Rosalinda et al. found that the combination of AZD5363 + AZD8542 + Curcumin and AZD8542 + Curcumin + Resveratrol significantly inhibited proliferation, induced apoptosis, and reduced glioma sphere activity [121].

In summary, drug therapy is a traditional treatment method, and the development of antitumor drugs is a crucial aspect of pharmaceutical science. Although the combination of drugs can enhance the therapeutic effect, an increasing number of studies have found that drug therapy alone also poses numerous challenging issues in clinical practice. For example, drugs cannot effectively accumulate in tumor cells due to low BBB penetration, drug resistance in tumor tissues, and immune escape.

#### 8.1.1 Mediating the Mitochondrial Autophagy Pathway

The PI3K/Akt/mTOR, MAPK/mTOR, and AMPK/mTOR signaling pathways have been widely reported to activate mitochondrial autophagy [122, 123]. It was found that Metformin [116], Curcumin [124], Resveratrol [125], Mahanine [126], Phenethyl isothiocyanate [127], Berberine (BBR) [128], Kaempferol [118], and Pantoprazole (PPZ) [129], along with other drugs, can inhibit mitochondrial metabolism, induce mitochondrial apoptosis, reduce glioma cell activity, and decrease glioma cell proliferation by mediating mitochondrial pathways such as mTOR, NF‐κB, PI3K/Akt, MAPK, and AMPK. This leads to achieving antiglioma effects [130]. Moreover, targeting FAM72A to reduce its expression could decrease its regulation of mitophagy through the Pink1/Parkin signaling pathway, thereby inhibiting glioma progression. Second, increasing the expression of METTL3 via small molecules or gene editing technology could enhance mitophagy, improving the metabolic state of GSCs, and inhibiting their proliferation and renewal [75].

#### 8.1.2 Activation of Caspase 3 and Caspase 9

The imprisoned ONC201 [131], Curcumin [132], Dioscin [133], Resveratrol [134], QCT [135], 4‐(N‐(S‐penicillamine acetylamino) phenylacetic acid (PENAO)) [136], PPZ [129] and BBR [137], and other drugs can trigger ROS production or loss of mitochondrial membrane potential, inducing mitophagy. Cytochrome c released from the cytoplasm forms a complex with Apaf‐1, promotes the activation of caspase 9, and then activates caspase 3 to induce apoptosis [138]. Among them, caspase 3 is considered the most important regulator of apoptosis, while caspase 9 is considered the primary regulator of mitochondria‐mediated apoptosis [139]. Therefore, inducing apoptosis by activating caspase 3 and caspase 9 is an effective therapeutic approach.

#### 8.1.3 Promoting Ferroptosis

Ferroptosis, a novel form of regulated cell death characterized by iron‐dependent lipid reactive oxygen species accumulation, has been implicated in glioma pathology. Zong et al. found that the ferroptosis inducer Erastin promotes ferroptosis in gliomas by inducing overexpression of heat shock protein 90 (Hsp90) and Drp1, and modulating the activity of acyl‐coenzyme A synthetase long‐chain family member 4 (Acsl4). The overexpression of Hsp90 and dephosphorylation of DRP1 alter mitochondrial morphology and enhance Acsl4‐mediated lipid peroxidation, facilitating ferroptosis [140]. Furthermore, activation of the Hsp90‐Acsl4 pathway enhances Erastin's anticancer effectiveness both in vitro and in vivo, providing a new and efficient ferroptosis‐mediated therapeutic approach for neurogliomas.

#### 8.1.4 Disruption of Mitochondrial Quality Control Processes

Mitochondrial quality control (MQC) enhancement, manifested as mitochondrial hyperpolarization, is a hallmark of glioblastoma multiforme. Thus, targeting MQC processes to disrupt mitochondrial homeostasis presents a promising therapeutic approach for GBM. Huang et al. discovered that the micropeptide MP31 induces a loss of MMP and triggers mitochondrial fission in GBM cells, while concurrently blocking mitophagy flux. This leads to the accumulation of damaged mitochondria, subsequent reactive oxygen species production, and DNA damage, thereby compromising MQC in GBM and inhibiting tumor growth [141]. Additionally, MP31 enhances the sensitivity of GBM cells to temozolomide in vitro and in vivo without affecting normal astrocytes or microglia. This suggests that its anticancer efficacy is achieved through the inhibition of protective mitochondrial functions.

#### 8.1.5 Affecting Mitochondrial DRP1

Resveratrol can be used to inhibit the expression of DRP1, a key protein in the mitochondrial fission process, reducing mitochondrial fission and thus inhibiting tumor cell invasion [142]. In addition, both chelerythrine (CHE) [143] and shikonin [144] can induce necrotic apoptosis through the formation of a complex mediated by mtROS‐mediated receptor‐interacting protein kinase 1‐receptor‐interacting protein kinase 3‐DRP1 (RIP1‐RIP3‐DRP1) [145], which promotes mitochondrial DRP1 translocation to enhance necrotic apoptosis. CHE and shikonin induce mitochondrial ROS production, mitochondrial depolarization, decreased ATP levels, and mitochondrial fragmentation. These events are crucial triggers for RIP1‐dependent activation of necrotic apoptosis.

#### 8.1.6 Disruption of Mitochondrial Transfer

Based on the role of mitochondrial transfer in GBM, the following therapeutic strategies can be proposed: First, targeting GAP43 to reduce its expression by developing small molecule inhibitors or RNA interference technology can decrease the promotion of mitochondrial transfer in astrocytes, slowing GBM progression [146]. Second, developing drugs that TNT formation could block mitochondrial transfer between cancer cells, reducing GBM cell proliferation and invasion. Additionally, supplementing mitochondrial protectants or antioxidants (such as NAC or CoQ10 [147]) can improve mitochondrial function in recipient cells, inhibiting tumor cell energy metabolism and further limiting their proliferation. These strategies aim to inhibit GBM progression by disrupting mitochondrial transfer mechanisms.

#### 8.1.7 Others

In addition to the aforementioned mechanisms, Mahanine, Dioscin, and BBR can also induce DNA damage to trigger cell cycle arrest, thereby inhibiting glioma migration and invasion. Cannabidiol can disrupt normal plasma membrane stability by influencing GBM lipid metabolism homeostasis, leading to increased phagocytosis of tumor cells by macrophages and demonstrating anti‐GBM effects [148]. The use of antioxidants, such as N‐acetylcysteine [149] and vitamin E [150], can effectively reduce intracellular ROS levels, protecting GBM cells from oxidative stress damage and improving cellular function. Interestingly, Dong et al. also found that both GSCs and differentiated glioblastoma cells exhibit robust circadian rhythms [151]. GSCs depend on the core clock transcription factors BMAL1 and CLOCK for optimal cell growth. By targeting BMAL1 and CLOCK in GSCs, it is possible to weaken mitochondrial metabolic function, reduce the expression of tricarboxylic acid cycle enzymes, and disrupt the growth and self‐renewal of GSCs [151, 152]. Therefore, targeting GSCs by disrupting the circadian clock provides a novel approach for antiglioblastoma treatment. Moreover, developing small molecule drugs or gene‐editing tools (such as CRISPR/Cas9 [153]) to target specific mtDNA mutations could help repair or replace mutated mtDNA, restoring normal mitochondrial function. Second, using antioxidants to reduce oxidative stress caused by mtDNA mutations could protect both tumor cells and their microenvironment. Additionally, metabolic interventions such as ketogenic diets [154] or caloric restriction [155] can alter tumor cell metabolic pathways, reducing their reliance on glycolysis and inhibiting cell proliferation.

### 8.2 Joint Nanotargeting Technologies

The most challenging issue facing immunotherapy is the accurate targeting of drugs. The presence of the BBB severely hampers the effectiveness of intracerebral drug delivery. Even when the drug manages to penetrate the BBB, it encounters challenges in accumulating within brain tumors, leading to poor therapeutic outcomes [156]. Therefore, renewed efforts to optimize BBB penetration techniques and develop BBB penetrators and drug delivery techniques that bypass the BBB are the focus of current GBM therapeutic research [103]. Several studies have shown that modern nanodrug delivery technologies and targeting peptides for mitochondria can achieve more effective drug delivery, deeper tissue penetration, and lower off‐target rates [103, 157].

Encapsulating drugs in nanocarriers can help them cross the BBB, reduce drug release in the body, and enhance drug accumulation in tumor tissues, thereby improving therapeutic efficacy. Fu et al. [158] and Jhaveri et al. [159] demonstrated that loading Resveratrol into polyethylene glycolized liposomes can overcome the disadvantage of RES as a free drug, enabling targeted delivery to tumor tissues. Chen et al. designed a nitric oxide‐driven nanomotor loaded with the glycolysis inhibitor Lonidamine to penetrate the BBB and induce an antitumor immune response in glioblastoma in a preclinical model [160].

In recent years, there has been an increasing amount of research on nanoparticles that aims to enhance drug efficacy by targeting specific subcellular organelle structures. Grasso et al. found that when the natural antioxidant ferulic acid was loaded onto a nanostructured lipid carrier (NLC), it inhibited cell proliferation and induced cellular effects more significantly than that of blank NLC and free ferulic acid, which shows that nanoparticles can synergize with loaded drugs to enhance antitumor therapeutic effects [161]. Sharma et al. proposed a novel dendrimer coupled with the TSPO ligand 5,7‐dimethylpyrazolo[1,5‐α]pyrimidine‐3‐acetamide, which can be used in glioblastoma to achieve tumor‐associated macrophage‐specific targeting in glioblastoma and can be further modified to target specific intracellular compartments for organelle‐specific drug delivery [162].

Currently, the primary optimization directions for nanocarriers involve continuously updating the structure of nanomaterials and nanocarriers to enhance loading efficiency, as well as improving the more precise targeting effect of nanocarriers. Targeting peptides are easy to synthesize, biocompatible, and highly absorbed in vivo. Nanocarriers modified with mitochondrial targeting peptides can further enhance the precision of drug targeting [163]. Nam et al. designed peptides with cyclohexyl residues that exhibit efficient cell permeation and mitochondrial localization. This design can promote drug selectivity and robust targeted delivery while minimizing off‐target effects [157].

Commonly used mitochondria‐targeting peptides include Szeto–Schiller peptide and mitochondria‐targeted sequence peptides (MTS) [164]. Kuang et al. developed SS‐31 peptide‐coupled nanoparticles. SS‐31 peptide‐modified nanoparticles demonstrated faster internalization than unmodified nanoparticles in mitochondria‐targeting studies, facilitating mitochondria‐targeted drug delivery [165]. Wei et al. proposed multifunctional polymeric micelles, P‐D‐MTS and P‐D‐R8MTS, based on the modification of prodrug‐loaded MTS. In vivo experiments in tumor‐bearing mice revealed that the mitochondria‐targeting sequences, along with cell‐penetrating peptides and copolymer couplers, effectively targeted mitochondria and significantly reduced tumor growth by inducing apoptosis [166]. Lin et al. [167] and Klimpel et al. [168] designed and analyzed bifunctional hybrid peptides consisting of MTS linked to a cell‐penetrating peptide. The bifunctional peptide hybrids were demonstrated to enhance the intracellular uptake of the cytostatic drug nitrogen mustard phenylbutyrate, showing effective mitochondrial targeting. This makes them strong candidates for further research in this important area. Thus, mitochondria‐targeted therapies, propelled by mitochondria‐targeted peptides, have faster and more precise targeting effects. In conclusion, the functionalization of nanocarriers with mitochondria‐targeting peptides enhances the biocompatibility and targeting efficiency of nanocarriers. The utilization of dual‐targeting peptides and multifunctional hybridized peptides could be a breakthrough in enhancing the targeting accuracy of nanoparticles. Meanwhile, the therapeutic strategy of dual‐loading drugs into nanoparticles also enhanced the therapeutic efficacy.

In addition to mitochondria‐targeted peptides, other mitochondria‐targeted compounds can also be combined with nanocarriers to enhance therapeutic effects. Yang et al. constructed Tween80‐loaded paclitaxel liposomes co‐modified with folic acid and Berberine derivatives. The folic acid–modified liposomes can effectively target glioma cells, while Berberine's positive electropositive and lipophilic properties enable attraction by the mitochondrial potential and aggregation to the mitochondria [169]. This mechanism achieves mitochondrial targeting and induces apoptosis, significantly enhancing the therapeutic effect of chemotherapy. This approach offers a novel idea and method for the targeted treatment of glioma.

In conclusion, drug delivery strategies based on nanotechnology and mitochondrial targeting offer the advantages of precision, high efficiency, and reduced side effects. However, these technologies are still in the research stage, and further in vivo studies are needed to assess the potential toxicity of the designed nanocarriers in biological systems, their residence time in vivo, and the release mechanism of the nanocarriers [164]. In addition, extensive research and clinical trials are fully addressing the in vivo targeting accuracy and safety of nanocarriers in combination with mitochondria‐targeted peptides, as well as exploring other novel therapeutic applications such as acoustic dynamics and photodynamics. With the continuous development and application of nanotechnology precision targeting, it is believed that more nanotechnology‐based antiglioma diagnostic and therapeutic methods will be developed and applied in the future. This advancement is expected to improve treatment outcomes and enhance the quality of survival for glioma patients.

### 8.3 Combined Novel Therapeutic Approaches

The combination of targeted drug therapy with other novel therapeutic approaches can also improve antiglioma outcomes. Kang et al. demonstrated the therapeutic efficacy of using mitochondria‐targeted photosensitizers loaded with albumin nanoparticles in photodynamic antiglioma therapy. They achieved significant tumor suppression through bi‐selective photodynamic therapy using mitochondria‐targeted photosensitizers and fiber‐optic cannulas for malignant gliomas. The study suggests that combining targeted therapies with photodynamic therapy offers a promising treatment strategy for malignant brain tumors [170]. In addition, Francesca et al. found that Berberine treatment in combination with photodynamic therapy may have potential therapeutic effects against malignant gliomas [171].

The intelligent “all‐in‐one” nanosensitizer platform designed by Qu et al. incorporated the sonoactive chlorine e6 and the autophagy inhibitor hydroxychloroquine into angiopep‐2 peptide‐modified liposomes (ACHL) [172]. ACHL was targeted by transient ultrasound. Microbubble disruption technology‐mediated BBB opening selectively accumulates in brain tumors within the optimal time window. The nanosensitizer responds to the second ultrasound stimulation by unloading hydroxychloroquine and generating ROS in glioma cells, simultaneously inducing mitophagy and apoptosis. This “all‐in‐one” nanosensitizer platform significantly inhibited the growth of xenograft tumors and prolonged the survival of tumor‐bearing mice. This is an intelligent strategic awareness that offers new insights into theranostics of brain tumors. In summary, combining drug therapy with other novel treatments is also a potential strategy to enhance therapeutic effects in anti‐glioma treatment.

## 9 Conclusions and Outlook

Effective diagnosis and treatment of gliomas have always been a clinical challenge. Studies on metabolic reprogramming, oxidative stress, kinetics, autophagy, and metastasis of mitochondria, along with other abnormal manifestations that promote the development of gliomas, have provided a clear idea and direction for glioma‐related treatment. However, the effects of mitochondrial kinetic changes and mitochondrial metastasis on gliomas need to be clarified through further experiments. In terms of therapeutic applications, many drugs targeting mitochondria have shown promising results in clinical trials for treating glioma. With the advancement of personalized, refined, and intelligent treatments, targeted therapy is being proposed as a strategy to enhance the precision of drug delivery. Therefore, targeting nanoparticle systems may be the most ideal choice for current glioma chemotherapy, but further experiments are needed to construct safe and reliable mitochondria‐targeted peptide‐functionalized nanosystems. In addition, the “all‐in‐one” nanosensitizer platform constructed by Qu et al. provides new insights into intelligent diagnosis and treatment through targeted nanoplatforms combined with physical ultrasound‐targeted microbubble destruction. With the continuous development and application of nanotechnology precision targeting, it is believed that more nanotechnology‐based antiglioma diagnostic and therapeutic methods will be developed and applied in the future. This advancement is expected to bring about better therapeutic effects and improve the quality of life for glioma patients.

## Conflicts of Interest

The authors declare no conflicts of interest.

## Data Availability

Data sharing not applicable to this article as no datasets were generated or analysed during the current study.
